# The Role of Sonic Hedgehog Pathway in the Development of the Central Nervous System and Aging-Related Neurodegenerative Diseases

**DOI:** 10.3389/fmolb.2021.711710

**Published:** 2021-07-08

**Authors:** Chen Yang, Yan Qi, Zhitang Sun

**Affiliations:** Department of Neurology, The Second Affiliated Hospital of Shanxi Medical University, Taiyuan, China

**Keywords:** Sonic hedgehog pathway, neurodegeneration, central nervous system, neurodegenerative diseases, therapeutic target

## Abstract

The Sonic hedgehog (SHH) pathway affects neurogenesis and neural patterning during the development of the central nervous system. Dysregulation of the SHH pathway in the brain contributes to aging-related neurodegenerative diseases such as Alzheimer’s disease, Parkinson’s disease, and amyotrophic lateral sclerosis. At present, the SHH signaling pathway can be divided into the canonical signaling pathway and non-canonical signaling pathway, which directly or indirectly mediates other related pathways involved in the development of neurodegenerative diseases. Hence, an in-depth knowledge of the SHH signaling pathway may open an avenue of possibilities for the treatment of neurodegenerative diseases. Here, we summarize the role and mechanism of the SHH signaling pathway in the development of the central nervous system and aging-related neurodegenerative diseases. In this review, we will also highlight the potential of the SHH pathway as a therapeutic target for treating neurodegenerative diseases.

## Introduction

**FIGURE 1:**
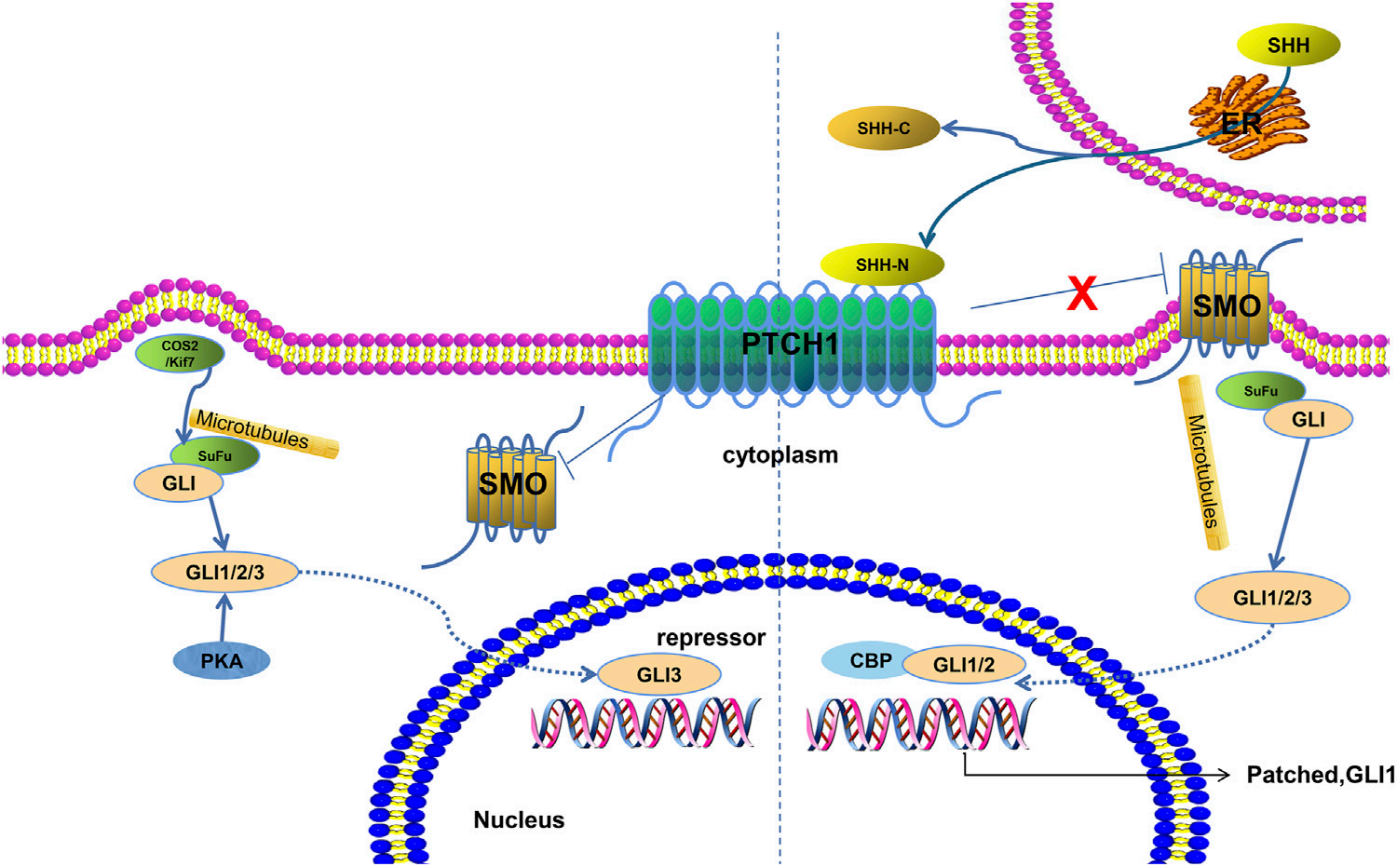
Sonic hedgehog signaling pathway. Two states are depicted. Under normal conditions, PTCH1 inhibits the activity of SMO protein, thereby inhibiting the downstream pathway. COS2/Kif7 and PKA play a negative regulatory role. When PTCH1 binds with SHH, the inhibition of SMO is released and the GLI protein and microtubule complex enters the nucleus to activate downstream target gene transcription.

Aging is a major risk factor for most neurodegenerative diseases such as Alzheimer’s disease, Parkinson’s disease, and amyotrophic lateral sclerosis (Hou et al., 2019). Most neurodegenerative diseases manifest in the elderly. However, there is still lack of effective treatment strategies against aging-related neurodegenerative diseases, which irreversibly progress and are related to much socioeconomic and personal costs (Ransohoff, 2016). Hence, novel and effective therapeutic strategies are desperately needed to combat these devastating diseases. The hedgehog (HH) gene was first found in wingless mutation of *Drosophila* (Lee et al., 1992). The secreted proteins encoded by the HH gene family act upon intracellular and distant cells to mediate relevant gene expression. In mammals, the HH family contains three members: desert hedgehog (DHH), Indian hedgehog (IHH), and Sonic hedgehog (SHH). Among them, SHH is widely expressed in human tissues and is involved in the development of the brain and spinal cord, axial bone, limbs, skin, hairs, teeth, cochlea, and lungs (Yang et al., 2018; Echevarría-Andino and Allen, 2020). The SHH signaling pathway can be classified into canonical and non-canonical pathways. The canonical SHH pathway primarily contains three components: a 12-domain transmembrane receptor (patched1, PTCH1), a seven-domain transmembrane receptor (Smoothened, SMO), and a GLIoma-associated oncogene homolog (GLI) family of transcription factors (GLI1, GLI2, and GLI3) (Espinosa-Bustos et al., 2019). The SHH ligand activates the SHH pathway and promotes the interaction between SMO and PTCH, thereby activating GLI protein (Figure 1). Particularly, SMO agonists seem to be promising for neurodegenerative diseases (Ruat et al., 2014). The interaction between activated GLI and CREB-binding protein (CBP) activates the transcription expression of downstream genes of SHH. Cell fusion inhibitor (SuFu), a key negative regulator in the SHH signaling pathway, inhibits GLI-mediated feedback activation by binding to GLI and acts as an adaptor protein that connects GLI protein and limb-dependent protease degradation pathway (Stone et al., 1999). The non-canonical SHH signaling pathway can be categorized into three types, as follows: 1) PTCH-mediated, 2) SMO-dependent/GLI-independent, and 3) SMO-independent GLI activation (Rimkus et al., 2016). Studies suggest that SHH exerts a decisive function in establishing the ventral spinal cord, inducing the basal lamina, and forming motor neurons and possesses various functions during the early stage of embryonic development such as forming a neural tube pattern, establishing the anterior–posterior axis and dorsal–ventral axis, and forming the body (Kvon et al., 2016). Dysregulation of SHH can induce neurodegenerative diseases (Patel et al., 2017). For instance, activation of the SHH pathway may protect dopaminergic neurons and attenuate inflammatory response through regulating the PI3K/Akt pathway in Parkinson’s disease (Shao et al., 2017). Meanwhile, it is cytoprotective against oxidative damage due to ALS (Peterson and Turnbull, 2012). Interference with the SHH pathway may sensitize HT22 cells, while augmentation of the SHH pathway may protect cells against hydrogen peroxide (H₂O₂) challenge (Peterson and Turnbull, 2012). Therefore, an in-depth study of this signaling pathway has important guiding significance and scientific value for understanding the pathogenesis, clinical diagnosis, and novel therapeutic strategies of neurodegenerative diseases. This mini-review will summarize the roles and mechanisms of the SHH pathway during the development of the central nervous system and neurodegenerative diseases.

### Role of Sonic Hedgehog Pathway in the Development of the Central Nervous System

During the development of the vertebrate central nervous system, SHH mediates ventral configurable, proliferative, and differentiated capacities of precursor cells (including telencephalon), thereby coordinating size, shape, and cell type (Ni et al., 2020). During the neocortex development, the SHH signaling pathway regulates proliferation of radial glial cells and intermediate precursor cells to maintain the proliferative capacity, differentiation, and survival of neocortex neurons (Komada, 2012). Dysregulation of SHH can lead to developmental disorders. In the central nervous system development of SHH gene knockout embryos of mice, early deficiency occurs in the midline structure and late deficiency includes the loss of distal limb structure, ciliary eye, the lack of ventral cell type in the neural tube, and the loss of spine and most ribs (Echevarría-Andino and Allen, 2020). In humans, SHH mutations have severe effects on the development of the forebrain and face, such as holoprosencephaly (Monteagudo, 2020). During the embryonic development, the interaction of bone morphogenetic protein (BMP) and SHH signaling pathway plays a decisive role in regulating the development of organ epithelial stem cells (Li et al., 2015). Moreover, SHH induces the development of other tissues or organs (Mangum et al., 2016). SHH deficiency affects the formation of the spinal cord structure as well as the development of the brain. The development of the vertebrate brain needs to create many types of cells at precise location and time, thereby forming functional connections. To produce the right number of cells, the brain must produce many precursors in a limited time. The SHH signaling pathway can control the brain developmental pattern as well as the size, shape, and direction of cells. First, SHH is expressed in the ventral side of the early embryonic development including the ventral hindbrain, midbrain, and forebrain, ensuring the normal constitution of the ventral structure. The early expression of SHH can induce forming aberrant brain structure. The cortex and basal ganglia are the main structures of the adult brain, which originate from embryonic telencephalon. SHH may induce diverse ventral neurons for the basal forebrain through GLI and regulate the size of the ventral midbrain (Ruiz i Altaba et al., 2002).

Recent studies show that SHH regulates the development of the nervous system through a synergistic effect with many factors. For example, the Nurr1 gene can inhibit cell apoptosis and inflammation, protect neurons, and cooperate with SHH to promote the development and maturation of neuron precursor cells (Palma et al., 2005). SHH can mediate the expression of Nkx2.1 in a GLI3-independent manner during embryonic neurogenesis, thus maintaining the normal activity of interneurons in the medial ganglionic eminence (MGE), which is the key determinant of the balance of excitation and inhibition in the postnatal cerebral cortex (Gulacsi and Anderson, 2006). SHH and basic fibroblast growth factor 8 (FGF-8) synergistically act on neural precursor cells and induce the expression of genes related to the development of dopamine neurons (Britto et al., 2002). Furthermore, this synergy can effectively induce human dental pulp stem cells to differentiate into nerve cells *in vitro* (Lambrichts et al., 2017). Experimental studies report that SHH cannot induce axon growth alone but can enhance retinoic acid receptor *β* 2 (RAR *β* 2), thereby stimulating the axon growth of neurons (So et al., 2006). Studies have shown that the nNOS–Sox2–SHH axis, as a new feedback compensation mechanism, participates in ischemic neuronal damage and plays a neuroprotective role in glutamate-induced excitotoxicity (Zhang et al., 2016). Furthermore, activation of the SHH signaling pathway can increase the expression of nerve growth factor (NGF), p75, and TrkA in the dorsal root ganglion (DRG) (Han et al., 2020).

SHH protein may produce amino and carboxyl terminal products by its own proteolysis. The amino terminal cleavage product (SHH-N) of SHH autoproteolysis can induce the differentiation of floor plate cells and motor neurons (Roelink et al., 1995). SHH-induced ventral neurons exhibit the gene expression pattern of lateral ganglion eminence, which is the embryonic structure derived from the striatum. The temporal regulation of SHH activity is indispensable in the sequential induction of basal telencephalon structures. Furthermore, the SHH signal may control cerebellum growth at different levels (Northcott et al., 2019). SHH is necessary for the proliferation of precursor cells of granular neurons. SHH that can be produced by Purkinje neurons induces the differentiation of Bergmann glial cells. *In vivo*, blocking SHH may lead to insufficient differentiation of granular neurons and Bergmann glial cells and abnormal development of Purkinje neurons (Wang and Almazan, 2016). According to numerous research studies, SHH can mediate to produce oligodendrocyte precursors (Namchaiw et al., 2019; Starikov and Kottmann, 2020). By the chemical inhibitor or anti-SHH antibody, inhibiting SHH may inhibit the differentiation of oligodendrocytes (Yang et al., 2020).

Combining previous studies, the SHH pathway plays a key role in the development of the central nervous system. Due to the complexity of distinct regions in the central nervous system, mechanisms involving SHH for neural precursor cell growth, neuron formation, and spinal cord syndesmosis need to be further studied.

### Roles and Mechanism of Sonic Hedgehog Pathway in Neurodegenerative Diseases

#### Sonic Hedgehog Signaling Pathway in Alzheimer’s Disease

Alzheimer’s disease (AD) is the most common neurodegenerative disease in adults characterized by progressive and irreversible memory decline, which mainly manifests cognitive impairment, behavioral change, and dysfunction (Cortes-Canteli and Iadecola, 2020). Neuropathological features include progressive degeneration of cholinergic neurons in the cholinergic projection system of the forebrain, especially in the basal nucleus of Meynert. However, the specific pathogenesis of AD has not been elucidated. The aggregation of the amyloid *β* (Aβ) peptide that is produced by the hydrolysis of amyloid precursor protein, as the main component of senile plaques, plays an important function on AD development. Recent evidence has demonstrated that SHH may participate in the cell cycle of post-mitotic neurons, and abnormal cell cycle promotes Aβ-induced neuronal death (Vorobyeva and Saunders, 2018). Furthermore, blockage of the SHH pathway may reduce apoptosis of hippocampal neurons to improve spatial learning and memory capacity in AD mice (Li et al., 2020). Primary cilia are small and static microtubules and cell membrane protuberance expressed in most vertebrate cells (like cortical and hippocampal neurons). They can be modified by various receptor proteins and are of necessity upon particular pathway cascades (like the SHH signal). Destruction of ciliary structures and functions can lead to various ciliary diseases. Cognitive impairment is a common human ciliary disease and a symptom of AD. A study has shown that the Aβ peptide can change the structure of primary cilia and damage the SHH signaling pathway via inhibiting the expression of Ptc1 and Gli1 in glial precursor cells (Chen et al., 2020).

Under normal circumstances, high levels of PTCH1 induced by SHH and relevant transcription factor GLI1 are related to the generation of specific neuronal progeny. However, their role in neural stem cells of AD remains unclear. Glycerylphosphorylcholine (GPC), a common choline compound and acetylcholine precursor in the brain, enhances memory and cognitive function in AD (Lee et al., 2017). The abnormal loss of GPC caused by out of control of the PTCH1/GLI1 signal may be one of the reasons for the cognitive impairment of the brain in AD (Moghadam et al., 2009). In the ventromedial cells of the developing central nervous system, SHH has been shown to affect the proliferation, differentiation, survival, and apoptosis of precursors. Transplantation of neural progenitor cells and cholinergic cells derived from mouse embryonic stem cells can promote the behavioral recovery of the AD rodent model (Reilly et al., 2002). Reilly et al. found that SHH exerts an important function on cholinergic neuron development, and its receptor PTCH1 is specifically expressed in cholinergic neurons of the adult rat basal forebrain, suggesting the therapeutic value of SHH in AD (Town et al., 2009). In the screening of genes regulated by the SHH signal in mouse limbs, the loss of GLI3 transcription factor may promote the up-regulation of PITRM1, a zinc metal endopeptidase that is related to AD and mitochondrial peptide degradation (Noguchi et al., 2015). However, some studies demonstrate that the SHH signal may induce AD. As an example, through establishing the “Aβ → Id1 → HIF-1 → SHH” signal cascade reaction in the rat cortex, Hong et al. found that SHH may be partly involved in the neurotoxicity of the Aβ peptide (Hung et al., 2016). Li et al. found that protease nexin-1 (PN-1) could reduce apoptosis of hippocampal neurons and improve spatial learning and memory ability by blocking the SHH pathway, thereby playing a protective role in AD (Li et al., 2020).

Collectively, the SHH pathway exerts an important function upon the neuronal activity for AD. Due to the complexity of the mechanism of the SHH pathway, its specific role in the pathogenesis of AD needs to be further explored, thereby providing the theoretical basis for the early intervention and therapy of AD.

#### Sonic Hedgehog Signaling Pathway in Parkinson’s Disease

The most important pathological change of Parkinson’s disease is the degeneration and death of dopaminergic neurons in the substantia nigra and striatum, which leads to the significant decrease of dopamine content in the substantia nigra and striatum pathway. As confirmed, SHH can suppress midbrain dopaminergic neuron deaths that are mediated via neurotoxin n-methyl-4-phenylpyridine (MPP+) (Ugbode et al., 2017). Intracerebral injection of SHH-N improves the motor function of rats with Parkinson’s disease and increases tyrosine hydroxylase immunoreactive neuron expression for the striatum, demonstrating that SHH possesses a specific treatment foreground against Parkinson’s disease, and SHH may be used as a potential therapeutic agent for neurodegenerative diseases (Dass et al., 2002). The SHH pathway can also protect dopaminergic neurons from oxidative stress by enhancing the activity of superoxide dismutase 1 (SOD1) (Ji et al., 2012). Xia Y-P et al. found that oxidative stress induces astrocytes to secrete endogenous SHH, and exogenous SHH may protect astrocytes by activating the PI3K/Akt/Bcl-2 pathway and reduce the apoptosis rate induced by H_2_O_2_ (Xia et al., 2012). The interaction between wingless-type MMTV integration site family (Wnt)/β-catenin and SHH-Smoothened pathways plays an important role in the development of dopaminergic progenitors and neurons (Luo and Huang, 2016).

Therefore, the SHH signaling pathway can protect dopaminergic neurons, which provides an important clue for developing the effective therapeutic strategies against Parkinson’s disease.

#### Sonic Hedgehog Signaling Pathway in Amyotrophic Lateral Sclerosis

Amyotrophic lateral sclerosis (ALS) is one of the most common motor neuron diseases (Chiò et al., 2020). The loss of the upper and lower motor neurons is the main aspect of ALS. The average course of ALS is only 3–5°years. Nevertheless, there are no effective therapeutic strategies (Turner et al., 2020). Most scholars believe that ALS is a complex disease caused by the interaction of multiple factors and genes. The main pathogenic theories include glutamate excitotoxicity, oxidative stress, protein misfolding, mitochondrial changes, axonal transport disorder, neuroinflammation, exogenous toxin and virus infection, gene mutation, abnormal RNA metabolism, etc. (Bennett et al., 2019; Calió et al., 2020; Harrison and Rafuse, 2020; Herrando-Grabulosa et al., 2021). However, little is known about its etiology, and there is no effective treatment for ALS. SHH is an essential morphological gene during the development of nervous systems. SHH exerts a critical function on promoting cell proliferation and differentiation, maintaining the integrity of blood–brain barrier, and protecting cells from oxidative stress (Xia et al., 2012). There is evidence that SHH exerts a key function on ALS (Lin et al., 2020). A study found that the decrease in the expression of cellular retinoic acid–binding protein 1 (CRABP1) is correlated with motor neuron degeneration (Lin et al., 2020). SHH may elevate CRABP1 expression through mediating GLI1, which involves specific chromatin remodeling (Lin et al., 2020). Because purmorphamine acts on Smoothened, downstream of SHH and its receptor Patched, the inhibitory action is downstream of Smoothened (Drannik et al., 2017). With purmorphamine, the induction activity of cerebrospinal fluid (CSF) in the control group is significantly enhanced, while there is no significant change in ALS, suggesting that the SHH signal may be inhibited in the CSF of ALS (Drannik et al., 2017). This proves that the SHH signal in the CSF of patients with ALS is damaged. The SHH signaling pathway may participate in the progression of ALS via interaction with other signaling pathways. For example, a study found that, in the spinal cord of mutant superoxide dismutase mice, the key markers of Notch signaling pathway change with age, and this trend is positively correlated with the change of the SHH signaling pathway, indicating that the association of these two pathways *in vivo* may contribute to dysfunction and death of motor neurons in ALS (Ma et al., 2017).

Collectively, targeting the SHH pathway could provide a novel insight into the therapeutic strategies against ALS. However, more cellular experiments and animal models should be performed to investigate the effects of SHH pathway on ALS progression.

## Discussion

The neurodegenerative disease represents the most common aging-related disease in the central nervous system. It is pathologically characterized by ion metabolism disorder, protein toxicity, oxidative stress, and neurotransmitter deficiency. Although numerous studies have been performed to elucidate the pathological mechanism of neurodegenerative diseases, the main reasons are still elusive. The aging population and the lack of effective therapeutic strategies make the prevention and treatment of neurodegenerative diseases face great challenges and bring heavy burden to the society, families, and individuals. The repair of central and peripheral nerve injury requires differentiating neural stem cells from functional neural cells. SHH may maintain neural stem cells and promote the formation of oligodendrocytes. This mini-review has summarized that the SHH signal exerts a key function on neurodegenerative diseases and becomes a key factor in the normal development of the vertebrate central nervous system. The SHH signaling pathway not only is a carrier of intracellular signal transmission but also participates in the normal development and differentiation of nerve cells. However, the abnormal SHH signaling pathway can lead to a variety of nervous system diseases. Although a lot of scientific research on SHH has been carried out in the past decades, many problems remain unsolved. In the future studies of patients with neurodegenerative diseases or animal models of neurological disorders, manipulation of SHH signaling by pharmacological or molecular approaches will improve our understanding of neurodegenerative diseases and may guide the way to new strategies for prevention and treatment.

## Future Perspectives

Despite many scientific studies on SHH in the past few decades, there are still many problems that have not been solved. New animal experimental models and technical means, gene, transcription, and protein detection technologies are increasingly improving, which may provide conditions for humans to conquer neurodegenerative diseases. Therefore, it is very important to deeply study the role and specific molecular mechanism of the SHH signaling pathway in the development of the central nervous system and neurodegenerative diseases. Moreover, exploring specific diagnostic and prognostic biomarkers and finding new therapeutic targets will bring hope for the cure of neurodegenerative diseases.

## Abbreviations

Aβ: amyloid β; AD: Alzheimer’s disease; ALS: amyotrophic lateral sclerosis; BMP: bone morphogenetic protein; CBP: CREB-binding protein; CSF: cerebrospinal fluid; DHH: desert hedgehog; FGF-8: fibroblast growth factor 8; GLI: GLIoma-associated oncogene homolog; GPC: glycerylphosphorylcholine; HH: hedgehog; IHH: Indian hedgehog; NGF: nerve growth factor; PTCH1: patched1; SHH: Sonic hedgehog; SMO: Smoothened; SOD1: superoxide dismutase 1; SuFu: cell fusion inhibitor; RAR β 2: retinoic acid receptor β 2.

## References

[B1] Bennett S. A. Tanaz R. Cobos S. N. Torrente M. P. (2019). Epigenetics in Amyotrophic Lateral Sclerosis: a Role for Histone post-translational Modifications in Neurodegenerative Disease. Translational Res. 204, 19–30. 10.1016/j.trsl.2018.10.002 PMC6331271 30391475

[B2] Britto J. Tannahill D. Keynes R. (2002). A Critical Role for Sonic Hedgehog Signaling in the Early Expansion of the Developing Brain. Nat. Neurosci. 5 (2), 103–110. 10.1038/nn797 11788837

[B3] Calió M. L. Henriques E. Siena A. Bertoncini C. R. A. Gil-Mohapel J. Rosenstock T. R. (2020). Mitochondrial Dysfunction, Neurogenesis, and Epigenetics: Putative Implications for Amyotrophic Lateral Sclerosis Neurodegeneration and Treatment. Front. Neurosci. 14, 679. 10.3389/fnins.2020.00679 32760239 PMC7373761

[B4] Chen S.-D. Yang J.-L. Lin Y.-C. Chao A.-C. Yang D.-I. (2020). Emerging Roles of Inhibitor of Differentiation-1 in Alzheimer's Disease: Cell Cycle Reentry and beyond. Cells 9 (7), 1746. 10.3390/cells9071746 PMC7409121 32708313

[B5] Chiò A. Mazzini L. Mora G. (2020). Disease-modifying Therapies in Amyotrophic Lateral Sclerosis. Neuropharmacology 167, 107986. 10.1016/j.neuropharm.2020.107986 32062193

[B6] Cortes-Canteli M. Iadecola C. (2020). Alzheimer's Disease and Vascular Aging. J. Am. Coll. Cardiol. 75 (8), 942–951. 10.1016/j.jacc.2019.10.062 32130930 PMC8046164

[B7] Dass B. Iravani M. M. Jackson M. J. Engber T. M. Galdes A. Jenner P. (2002). Behavioural and Immunohistochemical Changes Following Supranigral Administration of Sonic Hedgehog in 1-Methyl-4-Phenyl-1,2,3,6-Tetrahydropyridine-Treated Common Marmosets. Neuroscience 114 (1), 99–109. 10.1016/s0306-4522(02)00214-2 12207958

[B8] Drannik A. Martin J. Peterson R. Ma X. Jiang F. Turnbull J. (2017). Cerebrospinal Fluid from Patients with Amyotrophic Lateral Sclerosis Inhibits Sonic Hedgehog Function. PLoS One 12 (2), e0171668. 10.1371/journal.pone.0171668 28170441 PMC5295673

[B9] Echevarría-Andino M. L. Allen B. L. (2020). The Hedgehog Co-receptor BOC Differentially Regulates SHH Signaling during Craniofacial Development. Development 147 (23). 10.1242/dev.189076 PMC7758635 33060130

[B10] Espinosa-Bustos C. Mella J. Soto-Delgado J. Salas C. O. (2019). State of the Art of Smo Antagonists for Cancer Therapy: Advances in the Target Receptor and New Ligand Structures. Future Med. Chem. 11 (6), 617–638. 10.4155/fmc-2018-0497 30912670

[B11] Gulacsi A. Anderson S. A. (2006). Shh Maintains Nkx2.1 in the MGE by a Gli3-independent Mechanism. Cereb. Cortex 16 (Suppl. 1), i89–i95. 10.1093/cercor/bhk018 16766713

[B12] Han L. Jiang J. Xue M. Qin T. Xiao Y. Wu E. (2020). Sonic Hedgehog Signaling Pathway Promotes Pancreatic Cancer Pain via Nerve Growth Factor. Reg. Anesth. Pain Med. 45 (2), 137–144. 10.1136/rapm-2019-100991 31792027

[B13] Harrison J. M. Rafuse V. F. (2020). Muscle Fiber-type Specific Terminal Schwann Cell Pathology Leads to Sprouting Deficits Following Partial Denervation in SOD1G93A Mice. Neurobiol. Dis. 145, 105052. 10.1016/j.nbd.2020.105052 32827689

[B14] Herrando-Grabulosa M. Gaja-Capdevila N. Vela J. M. Navarro X. (2021). Sigma 1 Receptor as a Therapeutic Target for Amyotrophic Lateral Sclerosis. Br. J. Pharmacol. 178 (6), 1336–1352. 10.1111/bph.15224 32761823

[B15] Hou Y. Dan X. Babbar M. Wei Y. Hasselbalch S. G. Croteau D. L. (2019). Ageing as a Risk Factor for Neurodegenerative Disease. Nat. Rev. Neurol. 15 (10), 565–581. 10.1038/s41582-019-0244-7 31501588

[B16] Hung Y.-H. Chang S.-H. Huang C.-T. Yin J.-H. Hwang C.-S. Yang L.-Y. (2016). Inhibitor of Differentiation-1 and Hypoxia-Inducible Factor-1 Mediate Sonic Hedgehog Induction by Amyloid Beta-Peptide in Rat Cortical Neurons. Mol. Neurobiol. 53 (2), 793–809. 10.1007/s12035-014-9046-5 25502463

[B17] Ji H. Miao J. Zhang X. Du Y. Liu H. Li S. (2012). Inhibition of Sonic Hedgehog Signaling Aggravates Brain Damage Associated with the Down-Regulation of Gli1, Ptch1 and SOD1 Expression in Acute Ischemic Stroke. Neurosci. Lett. 506 (1), 1–6. 10.1016/j.neulet.2011.11.027 22133807

[B18] Komada M. (2012). Sonic Hedgehog Signaling Coordinates the Proliferation and Differentiation of Neural Stem/progenitor Cells by Regulating Cell Cycle Kinetics during Development of the Neocortex. Congenit. Anom. (Kyoto) 52 (2), 72–77. 10.1111/j.1741-4520.2012.00368.x 22639991

[B19] Kvon E. Z. Kamneva O. K. Melo U. S. Barozzi I. Osterwalder M. Mannion B. J. (2016). Progressive Loss of Function in a Limb Enhancer during Snake Evolution. Cell 167 (3), 633–642.e611. 10.1016/j.cell.2016.09.028 27768887 PMC5484524

[B20] Lambrichts I. Driesen R. B. Dillen Y. Gervois P. Ratajczak J. Vangansewinkel T. (2017). Dental Pulp Stem Cells: Their Potential in Reinnervation and Angiogenesis by Using Scaffolds. J. Endodontics 43 (9s), S12–s16. 10.1016/j.joen.2017.06.001 28781091

[B21] Lee J. J. von Kessler D. P. Parks S. Beachy P. A. (1992). Secretion and Localized Transcription Suggest a Role in Positional Signaling for Products of the Segmentation Gene Hedgehog. Cell 71 (1), 33–50. 10.1016/0092-8674(92)90264-d 1394430

[B22] Lee S. H. Choi B. Y. Kim J. H. Kho A. R. Sohn M. Song H. K. (2017). Late Treatment with Choline Alfoscerate (L-alpha Glycerylphosphorylcholine, α-GPC) Increases Hippocampal Neurogenesis and Provides protection against Seizure-Induced Neuronal Death and Cognitive Impairment. Brain Res. 1654 (Pt A), 66–76. 10.1016/j.brainres.2016.10.011 27765578

[B23] Li J. Feng J. Liu Y. Ho T.-V. Grimes W. Ho H. A. (2015). BMP-SHH Signaling Network Controls Epithelial Stem Cell Fate via Regulation of its Niche in the Developing Tooth. Developmental Cel 33 (2), 125–135. 10.1016/j.devcel.2015.02.021 PMC4406846 25865348

[B24] Li X.-L. Wang P. Xie Y. (2020). Protease Nexin-1 Protects against Alzheimer's Disease by Regulating the Sonic Hedgehog Signaling Pathway. Int. J. Neurosci. 2020:1–10. 10.1080/00207454.2020.1773821 32449865

[B25] Lin Y.-L. Lin Y.-W. Nhieu J. Zhang X. Wei L.-N. (2020). Sonic Hedgehog-Gli1 Signaling and Cellular Retinoic Acid Binding Protein 1 Gene Regulation in Motor Neuron Differentiation and Diseases. Ijms 21 (11), 4125. 10.3390/ijms21114125 PMC7312406 32527063

[B26] Luo S. X. Huang E. J. (2016). Dopaminergic Neurons and Brain Reward Pathways. Am. J. Pathol. 186 (3), 478–488. 10.1016/j.ajpath.2015.09.023 26724386 PMC4816693

[B27] Ma X. Drannik A. Jiang F. Peterson R. Turnbull J. (2017). Crosstalk between Notch and Sonic Hedgehog Signaling in a Mouse Model of Amyotrophic Lateral Sclerosis. Neuroreport 28 (3), 141–148. 10.1097/wnr.0000000000000725 27984541 PMC5434959

[B28] Mangum R. Varga E. Boué D. R. Capper D. Benesch M. Leonard J. (2016). SHH Desmoplastic/nodular Medulloblastoma and Gorlin Syndrome in the Setting of Down Syndrome: Case Report, Molecular Profiling, and Review of the Literature. Childs Nerv Syst. 32 (12), 2439–2446. 10.1007/s00381-016-3185-0 27444290

[B29] Moghadam F. H. Alaie H. Karbalaie K. Tanhaei S. Nasr Esfahani M. H. Baharvand H. (2009). Transplantation of Primed or Unprimed Mouse Embryonic Stem Cell-Derived Neural Precursor Cells Improves Cognitive Function in Alzheimerian Rats. Differentiation 78 (2-3), 59–68. 10.1016/j.diff.2009.06.005 19616885

[B30] Monteagudo A. (2020). Holoprosencephaly. Am. J. Obstet. Gynecol. 223 (6), B13–b16. 10.1016/j.ajog.2020.08.178 33168217

[B31] Namchaiw P. Wen H. Mayrhofer F. Chechneva O. Biswas S. Deng W. (2019). Temporal and Partial Inhibition of GLI1 in Neural Stem Cells (NSCs) Results in the Early Maturation of NSC Derived Oligodendrocytes *In Vitro* . Stem Cel Res Ther 10 (1), 272. 10.1186/s13287-019-1374-y PMC6712625 31455382

[B32] Ni Y. Chen D. Jiang Y. Qiu D. Li W. Li H. (2020). The Regenerative Potential of Facial Nerve Motoneurons Following Chronic Axotomy in Rats. Neural Plasticity 2020, 1–9. 10.1155/2020/8884511 PMC7416273 32802043

[B33] Noguchi K. K. Cabrera O. H. Swiney B. S. Salinas-Contreras P. Smith J. K. Farber N. B. (2015). Hedgehog Regulates Cerebellar Progenitor Cell and Medulloblastoma Apoptosis. Neurobiol. Dis. 83, 35–43. 10.1016/j.nbd.2015.08.020 26319366 PMC4674325

[B34] Northcott P. A. Robinson G. W. Kratz C. P. Mabbott D. J. Pomeroy S. L. Clifford S. C. (2019). Medulloblastoma. Nat. Rev. Dis. Primers 5 (1), 11. 10.1038/s41572-019-0063-6 30765705

[B35] Palma V. Lim D. A. Dahmane N. Sánchez P. Brionne T. C. Herzberg C. D. (2005). Sonic Hedgehog Controls Stem Cell Behavior in the Postnatal and Adult Brain. Development 132 (2), 335–344. 10.1242/dev.01567 15604099 PMC1431583

[B36] Patel S. S. Tomar S. Sharma D. Mahindroo N. Udayabanu M. (2017). Targeting Sonic Hedgehog Signaling in Neurological Disorders. Neurosci. Biobehavioral Rev. 74 (Pt A), 76–97. 10.1016/j.neubiorev.2017.01.008 28088536

[B37] Peterson R. Turnbull J. (2012). Sonic Hedgehog Is Cytoprotective against Oxidative challenge in a Cellular Model of Amyotrophic Lateral Sclerosis. J. Mol. Neurosci. 47 (1), 31–41. 10.1007/s12031-011-9660-x 21979788

[B38] Ransohoff R. M. (2016). How Neuroinflammation Contributes to Neurodegeneration. Science 353 (6301), 777–783. 10.1126/science.aag2590 27540165

[B39] Reilly J. O. Karavanova I. D. Williams K. P. Mahanthappa N. K. Allendoerfer K. L. (2002). Cooperative Effects of Sonic Hedgehog and NGF on Basal Forebrain Cholinergic Neurons. Mol. Cell Neurosci. 19 (1), 88–96. 10.1006/mcne.2001.1063 11817900

[B40] Rimkus T. Carpenter R. Qasem S. Chan M. Lo H.-W. (2016). Targeting the Sonic Hedgehog Signaling Pathway: Review of Smoothened and GLI Inhibitors. Cancers 8 (2), 22. 10.3390/cancers8020022 PMC4773745 26891329

[B41] Roelink H. Porter J. A. Chiang C. Tanabe Y. Chang D. T. Beachy P. A. (1995). Floor Plate and Motor Neuron Induction by Different Concentrations of the Amino-Terminal Cleavage Product of Sonic Hedgehog Autoproteolysis. Cell 81 (3), 445–455. 10.1016/0092-8674(95)90397-6 7736596

[B42] Ruat M. Hoch L. Faure H. Rognan D. (2014). Targeting of Smoothened for Therapeutic Gain. Trends Pharmacol. Sci. 35 (5), 237–246. 10.1016/j.tips.2014.03.002 24703627

[B43] Ruiz i Altaba A. Palma V. Dahmane N. (2002). Hedgehog-GLI Signaling and the Growth of the Brain. Nat. Rev. Neurosci. 3 (1), 24–33. 10.1038/nrn704 11823802

[B44] Shao S. Wang G.-L. Raymond C. Deng X.-H. Zhu X.-L. Wang D. (2017). Activation of Sonic Hedgehog Signal by Purmorphamine, in a Mouse Model of Parkinson's Disease, Protects Dopaminergic Neurons and Attenuates Inflammatory Response by Mediating PI3K/AKt Signaling Pathway. Mol. Med. Rep. 16 (2), 1269–1277. 10.3892/mmr.2017.6751 28627590 PMC5562000

[B45] So P.-L. Yip P. K. Bunting S. Wong L.-F. Mazarakis N. D. Hall S. (2006). Interactions between Retinoic Acid, Nerve Growth Factor and Sonic Hedgehog Signalling Pathways in Neurite Outgrowth. Developmental Biol. 298 (1), 167–175. 10.1016/j.ydbio.2006.06.027 16860305

[B46] Starikov L. Kottmann A. H. (2020). Diminished Ventral Oligodendrocyte Precursor Generation Results in the Subsequent Over-production of Dorsal Oligodendrocyte Precursors of Aberrant Morphology and Function. Neuroscience 450, 15–28. 10.1016/j.neuroscience.2020.05.027 32450295

[B47] Stone D. M. Murone M. Luoh S. Ye W. Armanini M. P. Gurney A. (1999). Characterization of the Human Suppressor of Fused, a Negative Regulator of the Zinc-finger Transcription Factor Gli. J. Cel Sci 112 (Pt 23), 4437–4448. 10.1242/jcs.112.23.4437 10564661

[B48] Town L. McGlinn E. Fiorenza S. Metzis V. Butterfield N. C. Richman J. M. (2009). The Metalloendopeptidase genePitrm1is Regulated by Hedgehog Signaling in the Developing Mouse Limb and Is Expressed in Muscle Progenitors. Dev. Dyn. 238 (12), 3175–3184. 10.1002/dvdy.22126 19877269

[B49] Turner M. R. Barohn R. J. Corcia P. Fink J. K. Harms M. B. Kiernan M. C. (2020). Primary Lateral Sclerosis: Consensus Diagnostic Criteria. J. Neurol. Neurosurg. Psychiatry 91 (4), 373–377. 10.1136/jnnp-2019-322541 32029539 PMC7147236

[B50] Ugbode C. I. Smith I. Whalley B. J. Hirst W. D. Rattray M. (2017). Sonic Hedgehog Signalling Mediates Astrocyte Crosstalk with Neurons to Confer Neuroprotection. J. Neurochem. 142 (3), 429–443. 10.1111/jnc.14064 28485896 PMC5575469

[B51] Vorobyeva A. G. Saunders A. J. (2018). Amyloid-β Interrupts Canonical Sonic Hedgehog Signaling by Distorting Primary Cilia Structure. Cilia 7, 5. 10.1186/s13630-018-0059-y 30140428 PMC6098584

[B52] Wang L.-C. Almazan G. (2016). Role of Sonic Hedgehog Signaling in Oligodendrocyte Differentiation. Neurochem. Res. 41 (12), 3289–3299. 10.1007/s11064-016-2061-3 27639396

[B53] Xia Y.-P. Dai R.-L. Li Y.-N. Mao L. Xue Y.-M. He Q.-W. (2012). The Protective Effect of Sonic Hedgehog Is Mediated by the Propidium Iodide 3-kinase/AKT/Bcl-2 Pathway in Cultured Rat Astrocytes under Oxidative Stress. Neuroscience 209, 1–11. 10.1016/j.neuroscience.2012.02.019 22402346

[B54] Yang C. Li X. Li Q. Li H. Qiao L. Guo Z. (2018). Sonic Hedgehog Regulation of the Neural Precursor Cell Fate during Chicken Optic Tectum Development. J. Mol. Neurosci. 64 (2), 287–299. 10.1007/s12031-017-1019-5 29285739

[B55] Yang W. Geng C. Yang Z. Xu B. Shi W. Yang Y. (2020). Deciphering the Roles of Caveolin in Neurodegenerative Diseases: The Good, the Bad and the Importance of Context. Ageing Res. Rev. 62, 101116. 10.1016/j.arr.2020.101116 32554058

[B56] Zhang D. Wang H. Liu H. Tao T. Wang N. Shen A. (2016). nNOS Translocates into the Nucleus and Interacts with Sox2 to Protect Neurons against Early Excitotoxicity via Promotion of Shh Transcription. Mol. Neurobiol. 53 (9), 6444–6458. 10.1007/s12035-015-9545-z 26607632

